# The identification of ᴅ-tryptophan as a bioactive substance for postembryonic ovarian development in the planarian *Dugesia ryukyuensis*

**DOI:** 10.1038/srep45175

**Published:** 2017-03-24

**Authors:** Kazuya Kobayashi, Takanobu Maezawa, Hiroyuki Tanaka, Hiroyuki Onuki, Yurie Horiguchi, Hiroshi Hirota, Tetsuo Ishida, Kihachiro Horiike, Yasutoshi Agata, Manabu Aoki, Motonori Hoshi, Midori Matsumoto

**Affiliations:** 1 Department of Biology, Faculty of Agriculture and Life Science, Hirosaki University, 3 Bunkyo-cho, Hirosaki, Aomori 036-8561, Japan; 2 Advanced Science Course, Department of Integrated Science and Technology, National Institute of Technology, Tsuyama College, 624-1 Numa, Tsuyama, Okayama 708-8509, Japan; 3 Department of Biochemistry and Molecular Biology, Shiga University of Medical Science, Tsukiwa-cho, Seta, Ohtsu, Shiga 520-2192, Japan; 4 RIKEN Genomic Sciences Center, 1-7-22 Suehiro-cho, Tsurumi-ku, Yokohama 230-0045, Japan; 5 Center for Integrated Medical Research, School of Medicine, Keio University, 35 Shinano-machi, Shinjuku-ku, Tokyo 160-8582, Japan; 6 Department of Biosciences and Informatics, Keio University, 3-14-1 Hiyoshi, Kouhoku-ku, Yokohama 223-8522, Japan

## Abstract

Many metazoans start germ cell development during embryogenesis, while some metazoans possessing pluripotent stem cells undergo postembryonic germ cell development. The latter reproduce asexually but develop germ cells from pluripotent stem cells or dormant primordial germ cells when they reproduce sexually. Sexual induction of the planarian *Dugesia ryukyuensis* is an important model for postembryonic germ cell development. In this experimental system, hermaphroditic reproductive organs are differentiated in presumptive gonadal regions by the administration of a crude extract from sexual planarians to asexual ones. However, the substances involved in the first event during postembryonic germ cell development, i.e., ovarian development, remain unknown. Here, we aimed to identify a bioactive compound associated with postembryonic ovarian development. Bioassay-guided fractionation identified ʟ-tryptophan (Trp) on the basis of electrospray ionization–mass spectrometry, circular dichroism, and nuclear magnetic resonance spectroscopy. Originally masked by a large amount of ʟ-Trp, ᴅ-Trp was detected by reverse-phase high-performance liquid chromatography. The ovary-inducing activity of ᴅ-Trp was 500 times more potent than that of ʟ-Trp. This is the first report describing a role for an intrinsic ᴅ-amino acid in postembryonic germ cell development. Our findings provide a novel insight into the mechanisms of germ cell development regulated by low-molecular weight bioactive compounds.

Many metazoans segregate primordial germ cells (PGCs) from somatic cells and initiate germ cell development during embryogenesis^1^. However, in some metazoans that possess pluripotent stem cells, such as hydras, planarians, annelids, and colonial ascidians, germ cell development from pluripotent stem cells and/or dormant PGCs occurs in the postembryonic stages^2^,^3^,^4^,^5^,^6^,^7^. The mechanism underlying postembryonic germ cell development is largely unknown.

Freshwater planarians (Platyhelminthes, Turbellaria, Seriata, and Tricladida) possess pluripotent stem cells, referred to as neoblasts. Neoblasts exist and proliferate within the mesenchymal space of the entire body of planarians, and differentiate into all types of planarian cell to support regeneration^8^,^9^,^10^,^11^,^12^,^13^,^14^,^15^,^16^,^17^. *Nanos* is well known as a conserved germline determinant in metazoans^3^,^4^,^6^,^18^,^19^,^20^. Meanwhile, it is expressed in PGCs after germline determination and is indispensable for the proliferation and maintenance of germ cells in mice^21^. Although a planarian *Nanos* homologue is not expressed in neoblasts, the expression of *Nanos* starts in PGCs within the presumptive gonadal regions in the postembryonic stages^4^,^6^,^22^,^23^. Knockdown of *Nanos* reduces the ability of germ cells in planarians to differentiate and proliferate. Therefore, planarian *Nanos* seems to be required for postembryonic germ cell development from the PGCs.

Some planarian species can reproduce asexually as well as sexually. Asexual worms regenerate lost body parts after fission without developing reproductive organs^24^. They seasonally develop hermaphroditic reproductive organs, i.e., sexual induction^25^,^26^,^27^,^28^. Although a seasonal change is mimicked by lowering the temperature under laboratory conditions, sexual induction is only occasionally triggered. Therefore, to study the cascade of molecular events during sexual induction, a reliable and reproducible assay system is required. It has been reported that asexual worms develop reproductive organs when they are fed minced sexually mature worms of the same or different planarian species^29^,^30^,^31^,^32^,^33^. This indicates that the sexually mature worms have a universal substance (or substances) that is responsible for sexual induction; herein referred to as a sex-inducing substance. To purify and identify such a substance, we established an assay system for sexual induction. Asexual *Dugesia ryukyuensis* worms of the OH strain were stimulated to develop reproductive organs by feeding them with sexually mature *Bdellocephala brunnea* worms^34^,^35^,^36^,^37^. We reported that the sex-inducing substance is a heat-stable and hydrophilic compound with an apparent molecular weight of less than 500^38^,^39^.

In asexual worms there are cell masses of female PGCs (ovarian primordia), but not male PGCs, in the presumptive gonadal regions, which are recognized by the expression of *Dr-nanos,* a *Nanos* homologue of *D. ryukyuensis*^23^. During sexual induction, the asexual worms first develop oogonia from the female PGCs and the oocytes start to differentiate. After ovarian growth, they develop male PGCs and the primordia of somatic reproductive organs such as yolk glands and copulatory apparatus from neoblasts. Finally, all reproductive organs become mature (Fig. 1). Thus, in the process of sexual induction, the sex-inducing substance triggers ovarian development from female PGCs as the first event. Without ovarian development, any other reproductive organs are never formed. This implies that the sex-inducing substance contains at least one bioactive substance associated with ovarian development.

In this study, we aimed to identify a sex-inducing substance that was associated with postembryonic ovarian development (female germ cell development). We identified ᴅ-tryptophan (ᴅ-Trp) as a sex-inducing substance. Our findings will open up new avenues of investigation into the role of low-molecular weight compounds for germ cell development.

## Results

### Identification of ʟ-Trp from *Bdellocephala brunnea*

**Figure 1:**
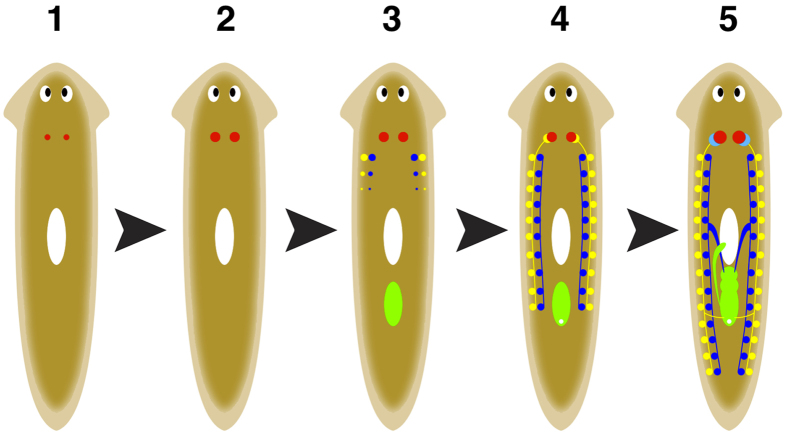
Five distinct stages of sexual induction. The development and topological positions of the reproductive organs are shown. The coloured regions correspond to the reproductive organs: red, ovary; aqua blue, seminal receptacle; blue, testis; yellow, yolk gland; green, copulatory apparatus with a genital pore. The white region represents the pharynx. The ovaries became sufficiently large to be externally apparent behind the head, although no oocytes or other reproductive organs were detectable in stage 1. Oocytes appeared in the ovaries, but other reproductive organs remained undetectable in stage 2. The primordial testes emerged and a copulatory apparatus became visible in the post-pharyngeal region in stage 3. Yolk gland primordia developed and spermatocytes appeared in the testes in stage 4. Mature yolk glands formed and many mature spermatozoa were detectable in the testes in stage 5.

**Figure 2:**
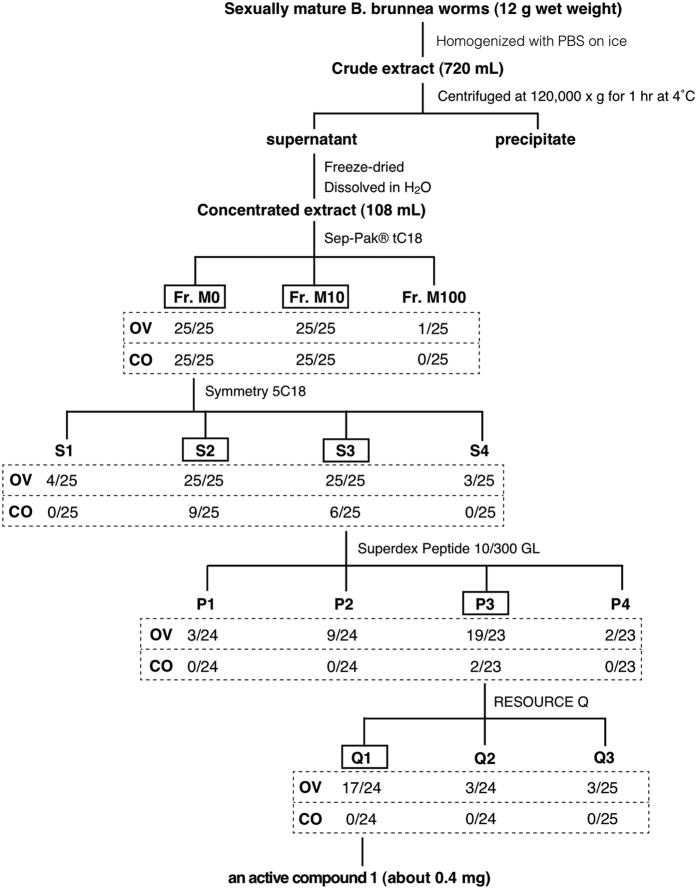
Overview of bioassay-guided fractionation of *Bdellocephala brunnea* extract. Extracts of sexually mature *B. brunnea* worms were applied to a Sep-Pak Light tC_18_ Cartridge and eluted with a three-step gradient of aqueous methanol. We named the resultant three fractions M0, M10, and M100. Fr. M0 was applied to a Symmetry 5C_18_ column and divided into four fractions (S1–S4) according to the retention time by elution with water. S3 was subsequently applied to a Superdex Peptide 10/300 GL column and divided into four fractions (P1–P4) according to the retention time by elution with water. Finally, P3 was applied to a RESOURCE Q column, washed with 20 mM Tris-HCl (pH 8.0), and developed with a linear gradient of 0–1 M NaCl contained in the same buffer. We called the wash fraction, the fraction eluted by 0–0.5 M NaCl, and the fraction eluted by 0.5–1.0 M NaCl Q1, Q2, and Q3, respectively. Sex-inducing activity (development of a pair of ovaries and copulatory apparatus) is shown in the boxes bounded by dotted lines below the label of each fraction. OV, the number of worms developing a pair of ovaries; CO, the number of worms developing copulatory apparatus. Fractions demonstrating strong sex-inducing activity at each purification step are highlighted in the box. Bioassay-guided fractionation led to the identification of the active compound.

For the isolation of the sex-inducing substance, 12 g in wet weight (about 210 worms) of pooled frozen *Bdellocephala brunnea* bodies was used for each purification step (Fig. 2). To produce the test food for the bioassay, we mixed each dried fraction in the purification steps with chicken liver homogenate, which is a food for planarian maintenance, and then freeze-dried the mixture. Under the conditions for our assay system^38^, asexual test worms were fed daily with a portion of the test food for 4 weeks. After feeding, the sex-inducing activity was evaluated by external observation. If the test food contains a sufficient quantity of the sex-inducing substance, a pair of ovaries and copulatory apparatus become externally visible in the asexual test worms^34^. The worms that develop externally visible ovaries and copulatory apparatus have already started to develop all reproductive organs within their bodies. In contrast, the worms that only develop externally visible ovaries have not yet developed the other reproductive organs (Fig. 1). We homogenized *B. brunnea* worms in phosphate-buffered saline (PBS) and obtained a cytosolic fraction after ultracentrifugation. The cytosolic fraction was applied to an octadecyʟ-silica (ODS) column and was eluted stepwise by changing the methanol concentration of the eluent (0, 10, and 100% (v/v)) (Fig. 2). We detected sex-inducing activity in the hydrophilic fractions (Frs) eluted with 0% (Fr. M0) and 10% (Fr. M10) methanol (v/v). All 25 asexual test worms fed with the test foods containing either Fr. M0 or Fr. M10 developed externally visible ovaries and copulatory apparatus. Previously, we showed that the expression of a marker gene for sexual maturation was higher in worms fed with Fr. M0 than in those fed with Fr. M10^38^. Therefore, we determined to use Fr. M0 for the isolation of the sex-inducing substance. Fr. M0 was further purified using several chromatographic methods (ODS column chromatography, gel filtration, and anion-exchange chromatography) guided by the bioassay system. Bioassay-guided isolation of the sex-inducing substance was carried out by estimating the sex-inducing activity at each purification step (as shown in the boxes bounded by dotted lines in Fig. 2). The sex-inducing activity (the ability to induce all reproductive organs in asexual test worms) gradually decreased after each purification step, but ovary-inducing activity persisted after the purification steps. This suggests that full sexual induction is orchestrated by several steps and is temporally regulated by multiple sex-inducing substances. Therefore, we attempted to identify a sex-inducing substance associated with ovarian development as a first step.

**Figure 3:**
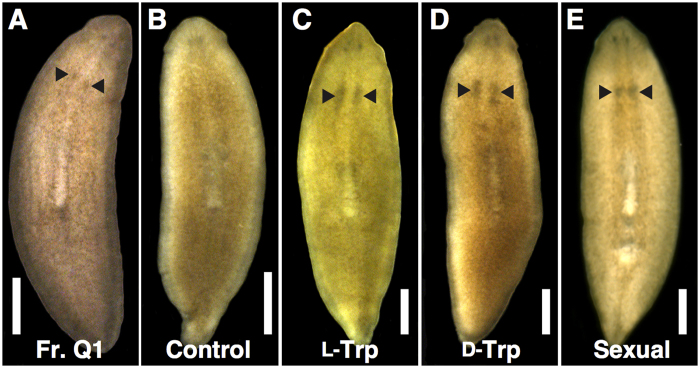
External morphology of Trp-fed worms. Ventral views of live worms are shown. The images are arranged with the anterior on the top. Asexual test worms were fed **(A)** Q1 obtained using RESOURCE Q identified as ʟ-Trp (Fr. Q1) for 4 weeks, **(B)** chicken liver homogenate for 4 weeks (control), **(C)** ʟ-Trp for 7 weeks, and **(D)** ᴅ-Trp for 7 weeks. **(E)** A sexually mature worm. The arrowheads represent a pair of ovaries. Scale bar, 2 mm.

The administration of the final fraction (Q1), obtained using RESOURCE Q, induced the development of a pair of externally visible ovaries in the asexual test worms (Figs 2 and 3A). This fraction was evaporated to dryness at 4 °C under reduced pressure to obtain compound **1** as a colourless powder (0.4 mg) (Fig. 2). Compound **1** exhibited a pseudo-molecular ion peak at *m*/*z* 205 in the electrospray ionization mass spectrum. In the proton nuclear magnetic resonance (^1^H NMR) spectrum, five aromatic and three aliphatic protons, along with a broad exchangeable proton, were observed. The ^13^C NMR spectrum showed 11 carbons: one carbonyl, eight aromatic, and two aliphatic carbons. Interpretation of conventional two-dimensional NMR spectra (correlation spectroscopy (COSY), hetero-nuclear multiple quantum coherence (HMQC), and hetero-nuclear multiple-bond connectivity (HMBC)) revealed that the planar structure of compound **1** was identical to that of commercial Trp. The *S*-configuration was determined using the circular dichroism (CD) spectrum, which showed a positive Cotton effect at 223 nm. Therefore, compound **1** was identified as ʟ-Trp (see Supplementary Fig. S1).

### Ovarian development in asexual worms by administration of ʟ-Trp

Preliminary experiments showed that the M10 fraction also contained Trp. To determine more precisely the Trp content of *B. brunnea*, we combined two fractions (M0 + M10). The combined fraction (M0 + M10) from *B. brunnea* (4 g wet weight) had sufficient activity to induce a pair of ovaries in 25 asexual worms^38^. The Trp content in the Fr. M0 + M10 from *B. brunnea* (4 g wet weight) was estimated to be 2,160 μg (Table 1).

To determine whether the administration of ʟ-Trp effectively induces ovarian development in asexual test worms, it was necessary to consider the endogenous ʟ-Trp content in the chicken liver homogenate used for the test food. The chicken liver homogenate did not have significant ovary-inducing activity (Fig. 3B), although a pair of small ovaries became visible in a few asexual test worms in this assay (Table 2). We estimated the Trp content of the chicken liver (4 g wet weight) to be 480 μg using the same measurement process used for *B. brunnea* (Table 1). Chicken liver homogenate (150 mg) was used as a test food for the 4-week bioassay. Based on the results shown in Table 1, the Trp content in the test food was estimated to be <20 μg. Therefore, we set the minimum amount of commercial ʟ-Trp in the test food to approximately 0.3 μg/worm/day (200 μg of ʟ-Trp for 25 asexual test worms for 4 weeks). Conversely, we set the maximum amount to approximately 3 μg/worm/day (2,000 μg of ʟ-Trp for 25 asexual test worms for 4 weeks), because the Trp content in *B. brunnea* was estimated to be 2,160 μg (Table 1). When asexual test worms were fed daily for 4 weeks with the test food containing commercial ʟ-Trp, they developed a pair of ovaries, similar to those developed using Fr. Q1 (Table 2). The ovaries developed further along the anterior–posterior axis of the worms as they were fed with ʟ-Trp over 7 weeks (Fig. 3C).

### Presence of ᴅ-Trp in *B. brunnea* and its effect on ovarian development

**Figure 4:**
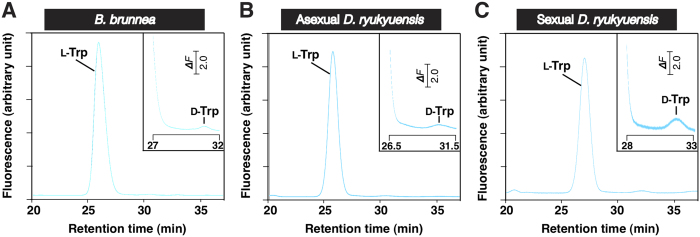
Chromatographic separation of ᴅ-Trp and ʟ-Trp in *Bdellocephala brunnea*, and in asexual and sexual *Dugesia ryukyuensis* worms. Trp from *B. brunnea*, and asexual and sexual *D. ryukyuensis* worms was treated with *o*-phthalaldehyde and *N*-acetyl-ʟ-cysteine, as described. Elution of the derivatives was monitored fluorometrically (excitation wavelength, 344 nm; emission wavelength, 443 nm). The retention times of the ʟ-Trp and ᴅ-Trp derivatives were 26.1 min (ʟ-Trp) and 30.7 min (ᴅ-Trp). The ratios of the ᴅ-Trp peak area to the ʟ-Trp peak area in **(A)** *B. brunnea*, **(B)** asexual, and **(C)** sexual *D. ryukyuensis* worms were 0.005, 0.002, and 0.014, respectively.

Preliminary experiments showed that the administration of either ᴅ-Trp or ʟ-Trp induced the development of a pair of ovaries in the asexual test worms. To confirm the presence of ᴅ-Trp in *B. brunnea*, we carried out diastereomeric derivatization of the Trp contained in *B. brunnea*, followed by reverse-phase high-performance liquid chromatography (HPLC), and found that the *B. brunnea* contained both ᴅ-Trp and ʟ-Trp in a ratio of 0.5% (w/w) (Fig. 4A). Based on the results shown in Table 1, the amount of ᴅ-Trp in the *B. brunnea* (4 g wet weight) was estimated to be 10 μg.

We attempted administration of ᴅ-Trp to asexual test worms. We set the standard amount for use at about 0.1 × 10^−1^ μg/worm/day (approximately 10 μg of ᴅ-Trp for 25 asexual test worms for 4 weeks), because the ᴅ-Trp content in 4 g (wet weight) of *B. brunnea* was estimated to be 10 μg (Table 1). Interestingly, the worms also developed a pair of ovaries following administration of commercial ᴅ-Trp at approximately 0.1 × 10^−3^ μg/worm/day to approximately 0.1 μg/worm/day for 4 weeks (Table 2). The ovaries induced by ᴅ-Trp for 4 weeks exhibited a similar external appearance to those by ʟ-Trp for 4 weeks and Fr. Q1. The ovaries induced by administration of either ʟ-Trp or ᴅ-Trp for 7 weeks exhibited a similar external appearance to those in sexually mature worms (Fig. 3D,E).

The effect on ovarian development of the administration of ᴅ-Trp or ʟ-Trp was investigated to determine the median effective dose (ED_50_). ED_50_ was calculated by non-linear regression using an in-house program, as described in Materials and Methods. Treatment with ᴅ-Trp resulted in ovary-inducing activity with an ED_50_ value of 0.55 ± 0.11 ng/worm/day; 500 times more potent than with ʟ-Trp, which had an ED_50_ of 0.30 ± 0.03 μg/worm/day (Table 2).

### Histological and *in situ* hybridization analyses of ovaries in Trp-fed worms

**Figure 5:**
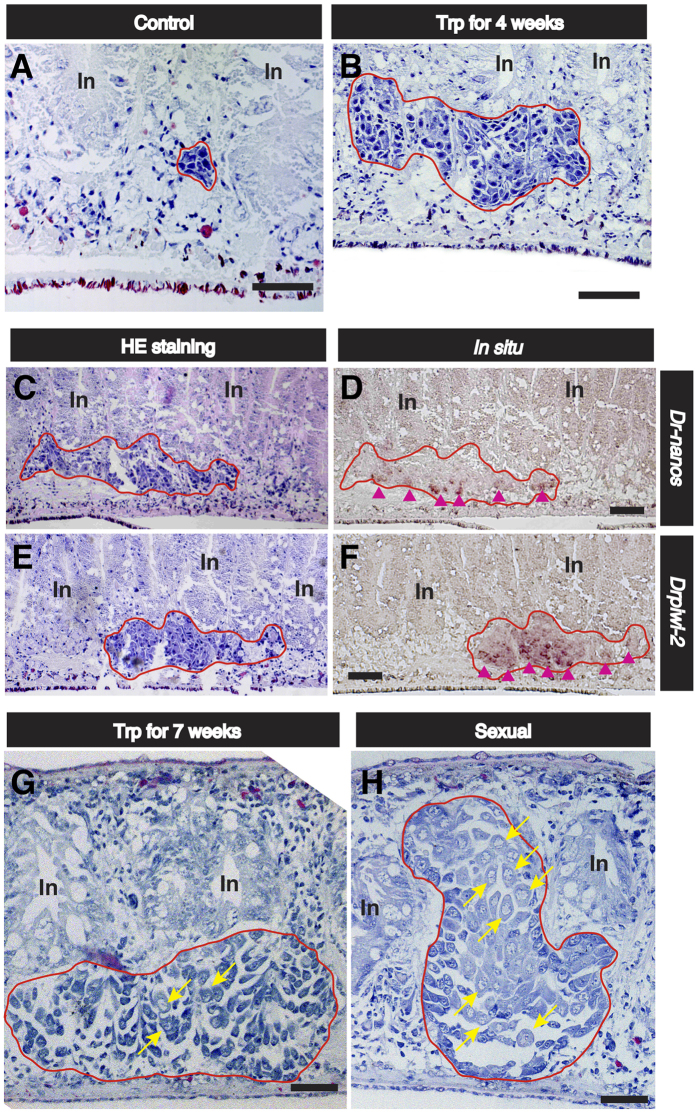
Ovarian morphology in Trp-fed worms. The ovaries induced by administration of ᴅ- and ʟ-Trp were morphologically indistinguishable. Embedded samples derived from worms fed with ᴅ-Trp (approximately 0.1 × 10^−1^ μg/worm/day) were sagittally sectioned. **(A–H)** Domains bounded by the red line are the female germ cell masses (ovaries). The dorsal sides are at the top. In, intestine. Scale bar, 100 μm. Hematoxylin and eosin (HE) stain images of **(A)** an ovary in a control worm fed with chicken liver homogenate, and **(B)** an ovary in the asexual test worms fed with ᴅ-Trp for 4 weeks. **(C–F)** An ovary in the asexual test worms fed with ᴅ-Trp for 4 weeks. Two neighbouring serial paraffin sections were used for staining with HE and *in situ* hybridization with each other. **(C,E)** HE staining. **(D)** *In situ* hybridization of *Dr-nanos*. **(F)** *In situ* hybridization of *Drpiwi-2*. The cells with the signals of *in situ* hybridization are represented by the magenta arrowheads. HE images of **(G)** an ovary in an asexual test worm fed with ᴅ-Trp for 7 weeks and **(H)** an ovary in a sexually mature worm. The cell indicated by the yellow arrow is an oocyte.

We performed histological analysis of the ovaries induced by administration of Trp. Asexual *D. ryukyuensis* worms have cell masses of female PGCs (ovarian primordia) in the presumptive ovarian regions, which are recognized by the expression of *Dr-nanos*^23^,^34^. Primordial ovaries were histologically recognized in the asexual test worms fed with dried chicken liver homogenate (a control) (Fig. 5A)^34^, although they were hardly visible externally (Fig. 3B). In contrast, ovaries developed by administration of ᴅ-Trp (approximately 0.1 × 10^−1^ μg/worm/day) for 4 weeks looked like proliferative cell masses of female PGC cells (Fig. 5B). To examine whether the cell masses were female PGC cells and/or oogonia, we performed *in situ* hybridization of *Dr-nanos*^23^ and *Drpiwi-2,* a *piwi* homologue in *D. ryukyuensis*^40^. In asexual worms, *Dr-nanos* was expressed in ovarian primordia, while *Drpiwi-2* was expressed in neoblasts as well as in ovarian primordia (see Supplementary Fig. S2). In the ovaries of sexual worms, *Dr-nanos* is strongly expressed in female PGCs but not in oogonia or oocytes^23^. In contrast, *Drpiwi-2* is strongly expressed in both female PGCs and oogonia but not in oocytes^40^. The expression of *Dr-nanos* was recognized in some cells around the bottom of the cell masses (Fig. 5C,D), whereas the expression of *Drpiwi-2* was recognized in almost all cells in the cell masses (Fig. 5E,F). This suggests that ovarian differentiation progressed to a slight degree from female PGC to oogonia in the cell masses induced by the administration of ᴅ-Trp. When the administration of ᴅ-Trp was extended until Week 7, the ovaries enlarged externally like those in sexually mature worms (Fig. 3C–E). Histological research revealed that an ovary induced by ᴅ-Trp had a few oocytes (Fig. 5G), whereas an ovary in a sexually mature worm had many oocytes (Fig. 5H), suggesting that Trp hardly affected oocyte maturation. However, this result indicates that at least some differentiation of oogonia occurred in the ovary induced by ᴅ-Trp. Testes, yolk glands, and copulatory apparatus were not recognized histologically. The ovaries induced by the administration of ᴅ- and ʟ-Trp were morphologically identical. Therefore, ᴅ- and ʟ-Trp act as sex-inducing substances that are associated with the postembryonic ovarian development required for the primary event of sexual induction.

### Analysis of Trp in *D. ryukyuensis* asexual and sexual worms

We estimated Trp content in 4 g (wet weight) each of asexual (approximately 2,000) and sexual (approximately 400) *D. ryukyuensis* worms. The Trp level was much higher in the sexual worms than in the asexual worms (approximately 25-fold per wet weight) (Table 1). Using the same method for the detection of ᴅ-Trp in *B. brunnea*, we performed diastereomeric derivatization of Trp from asexual and sexual worms, followed by reverse-phase HPLC. We found asexual worms contained ᴅ-Trp and ʟ-Trp in a ratio of 0.2% (w/w) (Fig. 4B), whereas sexual worms contained ᴅ-Trp and ʟ-Trp in a ratio of 1.4% (w/w) (Fig. 4C).

**Figure 6:**
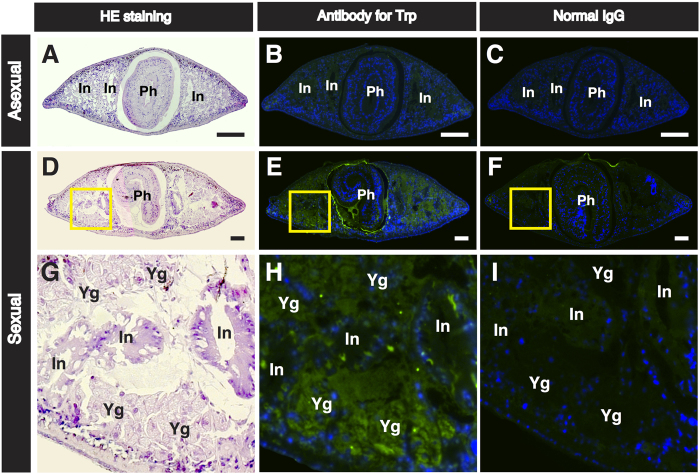
Immunofluorescence staining using a free ᴅʟ-Trp antibody in asexual and sexual worms. Transverse sections of asexual and sexual worms at the middle of the body (around the pharynx) were prepared. Three neighbouring serial paraffin sections were used for staining with hematoxylin and eosin (HE) and immunostaining with an antibody for free ᴅʟ-Trp and normal IgG, respectively. **(A,D,G)** Sections stained with HE. **(B,E,H)** Immunofluorescence micrographs using an antibody for free ᴅʟ-Trp. **(C,F,I)** Immunofluorescence micrographs using a normal IgG (control). The signals were visualized using the Alexa 488 (green) fluorophore. The nuclei were counterstained with Hoechst 33342 (blue), and the merged images are shown. The same settings were used to acquire all immunofluorescence images. **(A–C)** An asexual worm. **(D–F)** A sexual worm. **(G–I)** High magnification of squares bounded by the yellow line in **(D–F)**, respectively. In, intestine; Ph, pharynx; Yg, yolk gland. Scale bar, 500 μm.

To confirm whether sexual worms have organs/tissues capable of pooling a large amount of Trp, we carried out immunofluorescence staining using a polyclonal antibody for free ᴅʟ-Trp (Fig. 6). In the asexual worms, the intestine produced a weak immunofluorescence signal (Fig. 6A–C). In contrast, in the sexual worms an immunofluorescence signal was detected from a cell layer surrounding the pharynx (Fig. 6D–F), and in the yolk gland, which is a planarian-specific reproductive organ (Fig. 6G–I). In sexual worms, free Trp appears to be predominantly localized in the yolk glands, which are distributed over most of the body (Fig. 1). These immunohistochemical results are consistent with the quantitative biochemical results that show that sexual worms have higher levels of Trp than asexual worms (Table 1).

## Discussion

Information on low-molecular weight compounds required for postembryonic germ cell development in metazoans is scarce. Postembryonic germ cell development occurs when planarians switch from asexual to sexual reproduction. Interestingly, if asexual worms are fed with sexual ones, they develop hermaphroditic reproductive organs^29^,^30^,^31^,^32^,^33^. This suggests that sexual worms contain compound(s) called sex-inducing substance(s) that are involved in postembryonic germ cell development. In the sexual induction of *Dugesia ryukyuensis* asexual worms by the administration of sex-inducing substance(s) contained in the oviparous species *Bdellocephala brunnea*, differentiation of reproductive organs other than ovaries does not occur prior to ovarian development (Fig. 1). This indicates that at least one sex-inducing substance is involved in ovarian development. Sex-inducing activity that was able to induce the development of all reproductive organs in asexual worms diminished as the purification steps of the sex-inducing substances proceeded (Fig. 2). Instead, sex-inducing activity that was able to induce only ovaries (ovary-inducing activity) was detected in all the fractions assayed (Fig. 2). This suggests that full sexual induction is orchestrated by several steps and is temporally regulated by multiple sex-inducing substances. In this study, we aimed to identify a sex-inducing substance associated with postembryonic ovarian development (female germ cell development). We identified ᴅ-Trp and ʟ-Trp as the sex-inducing substances for ovarian development, although the ovaries remained immature (Figs 4A and 5). Asexual *D. ryukyuensis* worms have cell masses containing a small number of female primordial germ cells (PGCs) in presumptive ovarian regions^23^,^34^. Because the ovaries induced by Trp had at least a few oocytes, differentiation into oogonia from the female PGCs was possible. Therefore, Trp may play an important role in the proliferation of female PGCs and/or the differentiation of oogonia from the female PGCs. ʟ-Trp is the precursor for a variety of compounds such as ᴅ-Trp, serotonin (an important neuromediator), and kynurenine (an intermediary metabolite in a complex metabolic pathway that ends with niacin)^41^. In general, free ᴅ-amino acids are produced from free ʟ-amino acids by amino acid racemases^42^. The administration of a large amount of ʟ-Trp to asexual test worms might lead to an increase in metabolites, including ᴅ-Trp, produced in metabolic pathways derived from ʟ-Trp, and these metabolites may influence ovary development. Because the ovary-inducing activity by ᴅ-Trp was 500 times more potent than that by ʟ-Trp (Table 2), ᴅ-Trp must act as a principal bioactive compound in terms of ovarian development.

Based on the results shown in Table 1, the estimated Trp content in 12 g wet weight of *B. brunnea* was approximately 6 mg. However, the Trp content in Fr. Q1 was approximately 0.4 mg (Fig. 2), indicating that the remaining Trp from 12 g wet weight of *B. brunnea* dispersed into fractions other than Fr. Q1 during the purification process. As depicted in Fig. 2, the ovary-inducing activity of fractions other than Fr. Q1 may be attributed to Trp and/or other sex-inducing substances contained within these fractions.

ʟ-Trp is an essential amino acid that can only be derived from dietary proteins in animals^43^. Here, we showed that the yolk glands in the sexual *D. ryukyuensis* worms contained a large amount of ʟ-Trp (Table 1, Figs 4C and 6). A yolk gland is a reproductive organ filled with planarian nurse cells, namely yolk gland cells. Planarian eggs are called composite eggs (cocoons), which have several fertilized eggs and numerous yolk gland cells^44^,^45^,^46^. Levels of a yolk gland marker gene, *Dryg*^47^, were extremely high in fresh cocoons collected within a day of deposition (see Supplementary Fig. S3A). This suggests that the fresh cocoons still have intact yolk gland cells. We compared the amounts of proteinogenic amino acids in asexual worms, sexual worms, and fresh cocoons, and found that only Trp is prominently incorporated into the fresh cocoons (see Supplementary Table S1). This indicates that sexual planarians have the ability to selectively incorporate and pool Trp in their yolk glands.

We also found that the asexual worms (4 g wet weight; approximately 2000 worms) and the sexual worms (approximately 400 worms) contained ᴅ-Trp and ʟ-Trp in ratios of 0.2% and 1.4% (w/w), respectively (Fig. 4B,C). Based on the results shown in Table 1, the amounts of ᴅ-Trp in the asexual and sexual worms (4 g wet weight) were estimated to be 0.6 μg and 100 μg, respectively. Moreover, we expected that in the sexual worms the ᴅ-Trp would exist almost exclusively in the yolk glands. In general, biosynthesis and degeneration of ᴅ-amino acids are explained by the expression of amino acid racemases and ᴅ-amino aciᴅ-degrading enzymes^42^. Although ᴅ-amino acids are produced in most cases by racemization from their ʟ-forms by the action of racemases^48^, a tryptophan-specific racemase has not yet been isolated in any organism. In planarians, ᴅ-Trp may be produced by an unidentified tryptophan-specific racemase. A large amount of ʟ-Trp, pooled in yolk glands, is the perfect substrate for supplying ᴅ-Trp via the tryptophan-specific racemase. Alternatively, symbiotic and intestinal bacteria in planarians may produce ᴅ-Trp and supply it to planarian bodies. ᴅ-amino acid oxidase (DAO) orthologs that degrade neutral and basic ᴅ-amino acids (including ᴅ-Trp) have been found in several species from yeast to humans with a high degree of conservation^49^. Previously, we showed that Dr-DAO recombinant protein, which is coded by a *DAO* homolog gene (*Dr-DAO* in *D. ryukyuensis*) degraded ᴅ-Trp *in vitro*^50^. As expected, the expression of *Dr-DAO* in fresh cocoons was hardly detected (see Supplementary Fig. S3B). This suggests that the ᴅ-Trp in yolk glands escapes degradation by Dr-DAO. This is one of the reasons the sexual worms contained much larger quantities of ᴅ-Trp than the asexual worms. Substances contained in yolk gland cells are used as nutrients for embryogenesis^44^,^45^,^46^. Thus, the embryos may receive ᴅ-Trp. At the late stage of embryogenesis, the female PGCs appear, but ovarian development does not begin. After hatching, juveniles may utilize the ᴅ-Trp incorporated for ovarian development.

The expression of *Dr-DAO* in asexual worms was significantly higher than in sexual worms (see Supplementary Fig. S3B). Interestingly, knockdown of *Dr-DAO* led to ovarian development in asexual worms^50^. This implies that the increase in the concentration of ᴅ-Trp in the asexual worms was due to the decrease of DAO activity, resulting in ovarian development. In nature, environmental factors may trigger reproductive switching in *D. ryukyuensis*. In the switch from an asexual to a sexual state, those environmental factors could lead to accumulation of ʟ-Trp from foods, repression of Dr-DAO, and/or activation of an unidentified tryptophan-specific racemase in asexual worms, resulting in female PGC proliferation and differentiation caused by an increase in ᴅ-Trp. The change in Trp metabolism might be essential for the initiation of sexual induction in *D. ryukyuensis*.

Various ᴅ-amino acids have been found in archaea, yeasts, fungi, plants, insects, molluscs, and other eukaryotic organisms. ᴅ-amino acids play a key role in the regulation of many biological processes in living organisms^51^,^52^. For example, ᴅ-serine is the endogenous coagonist of *N*-methyl-ᴅ-aspartate (NMDA) receptors, which are important for excitatory synaptic transmission, and are involved in various processes, such as learning and memory retention^53^. ᴅ-Aspartate regulates steroidogenesis and spermatogenesis in vertebrate testes^54^,^55^. We do not yet know how ᴅ-Trp controls female PGC proliferation and differentiation in *D. ryukyuensis*. There are many reports of peptides, with bioactivity in various animals, which incorporate ᴅ-amino acids^56^,^57^. In gastropod mollusks, the tripeptide ʟ-Asn-ᴅ-Trp-ʟ-Phe-NH_2_ (NdWFamide) acts as a neuropeptide^58^,^59^,^60^,^61^,^62^. In general, it is thought that ᴅ-amino acids in proteins (peptides) are enzymatically converted from ʟ-amino acids after translation as a post-translational modification. Thus, it may be reasoned that free ᴅ-Trp would not be incorporated into proteins such as neuropeptides in the planarian bodies. In human leukocytes, ᴅ-Trp acts as a chemoattractant factor through a human niacin receptor^63^. There is no homolog gene of the niacin receptor GPCR109B in the expressed sequence tag (EST) database entry for *D. ryukyuensis*^64^, nor for the genome database entry for the planarian *Schmidtea mediterranea*^65^. It is possible that ᴅ-Trp functions via an unknown receptor in planarians. Our findings with regard to intrinsic ᴅ-Trp represent the first reported example of a ᴅ-amino acid that regulates ovarian development. The novel and common mechanism of ovarian development induced by ᴅ-Trp in metazoans may be elucidated in the near future.

## Materials and Methods

### Animals

*D. ryukyuensis* planarian worms of an exclusively asexual strain, the OH strain, were maintained at 20 °C in dechlorinated tap water and fed chicken liver once a week. The asexual worms of this strain were used as test animals. Wild populations of *B. brunnea*, an oviparous planarian, were collected near Yamagata City, Japan, frozen in liquid nitrogen, and stored at −80 °C for the source of sex-inducing substances. The sexual *D. ryukyuensis* worms were obtained by feeding asexual worms with *B. brunnea*, as described previously^34^. The sexual worms were then maintained under the same conditions (diet of chicken liver) as the asexual worms for approximately 1 year.

### Bioassay-guided fractionation of the aqueous extract from *Bdellocephala brunnea*

Fr. M0 from pooled frozen *B. brunnea* bodies was prepared as described previously^38^. Further bioassay-guided fractionation was performed using HPLC apparatus (ÄKTA explorer 10S controlled by UNICORN software version 4.11, Amersham). Fr. M0 was applied to a Symmetry 5C_18_ column (4.6 × 150 mm, Waters) equilibrated with H_2_O and isocratically eluted with H_2_O at a flow rate of 1 mL/min. The elution was monitored at 220 nm and divided into four fractions according to the retention time (5–17 min (S1), 17–22 min (S2), 22–40 min (S3), and 40–60 min (S4)). S3 was subsequently applied to a Superdex Peptide 10/300 GL column (Amersham) with H_2_O as the mobile phase at a flow rate of 1 mL/min. The peak areas within the particular retention time (5–15 min (P1), 15–20 min (P2), 20–25 min (P3), and 25–30 min (P4)) were collected separately and tested for sex-inducing activity. P3 was applied to a RESOURCE Q column (6 mL, Amersham) equilibrated with 20 mM Tris-HCl (pH 8.0); the column was washed with the same buffer (6 mL) and then developed with a linear gradient of 0–1 M NaCl contained in the same buffer at a flow rate 1.0 mL/min. Elution was monitored at 220 nm, and the wash fraction (4–10 min, Q1), 0–0.5 M NaCl (10–20 min, Q2), and 0.5–1.0 M NaCl (20–30 min, Q3) were collected separately. Fractions obtained using RESOURCE Q were further purified by passing them through a Superdex 75 10/300 GL column (Amersham) to remove buffer salts. The obtained fractions were freeze-dried for bioassay. Q1 was also used for NMR, CD and ESI-MS analyses.

### Bioassay

For the preparation of test food, dried fractions and commercial Trp were mixed with chicken liver homogenate and then freeze-dried. The asexual test worms were fed daily for 4–7 weeks using a previously described method^38^. To evaluate the ovarian induction in the asexual test worms, observations were performed under a binocular microscope (SZX9; Olympus Optics, Tokyo, Japan), paying close attention to the development of the ovaries and copulatory apparatus.

### NMR, CD and ESI-MS analyses of Q1 obtained using RESOURCE Q

NMR spectra were recorded at 25 °C with a Bruker AVANCE 500 spectrometer. For ^1^H NMR spectra, each aqueous fraction was concentrated to 300 μL at 4 °C under reduced pressure using a centrifugal evaporator, and then 33 μL of D_2_O was added. For two-dimensional NMR spectra, the samples were concentrated using a centrifugal evaporator to dryness at 4 °C and then dissolved in 300 μL of D_2_O. CD spectra were recorded at 20 °C with a JASCO J-820 spectropolarimeter by using a quartz cell with a path length of 2 mm. Mass spectra were obtained with a hybrid qTOF mass spectrometer (QSTAR, Applied Biosystems) equipped with an electrospray ion source (infusion injection mode; the samples were dissolved in acetonitrile/water (1:1 v/v) containing 0.1% formic acid).

### Estimation of Trp contents in Fr. M0 + M10

Each Fr. M0 + M10 from 4 g in wet weight of *B. brunnea*, chicken liver, and asexual and sexual *D. ryukyuensis* worms was applied to a C_18_ column (ODS-HG-5, Nomura Chemical), and the column was developed with 5% aqueous acetonitrile (v/v) by HPLC (JASCO 807-IT Integrator, JASCO PU-2089 Plus Quaternary Gradient Pump, and UV-2075 Plus Intelligent UV detector). We collected a single peak corresponding to commercial Trp and then confirmed the presence of Trp using a liquid chromatography-mass spectrometry apparatus equipped with an electrospray ionization ion source as a significant peak with m/z 205.0. To estimate Trp content in each Fr. M0 + M10 prepared from the samples, various known amounts of commercial Trp (0, 500, and 1000 μg) were subjected to the ODS-HG-5 column, the peak area corresponding to Trp was measured, and the calibration curve was established. The amount of Trp in each sample was then estimated from the calibration curve.

### Diastereomeric derivatization and HPLC of Trp enantiomers in *B. brunnea*, and asexual and sexual *D. ryukyuensis* worms

We examined the ᴅ-Trp content in Trp derived from Fr. M0 + M10 (Table 1) using a previously described method^66^ with slight modifications. Briefly, an aliquot of Trp derived from Fr. M0 + M10 (10–50 μL) was mixed with four volumes of methanol. After centrifugation for 10 min at 12,000 × *g* at 4 °C, a 60-μL aliquot of the supernatant was transferred to a 1.5-mL Eppendorf tube and dried at 60 °C under reduced pressure. The resultant residue was dissolved with 20 μL of 20 mM sodium borate (pH 8.0), and the insoluble material was removed by centrifugation. An aliquot of the supernatant (10 μL) was mixed with 0.5 μL of 50% methanol containing 19 mM *o*-phthalaldehyde and 15 mM *N*-acetyʟ-ʟ-cysteine, and was then incubated at room temperature (RT) for 2 min. The resultant amino acid derivatives were immediately separated using a Cosmosil 3C_18_ column (4.6 × 100 mm) (Nacalai Tesque, Kyoto, Japan).

### Histology

The worms were fixed in 10% formalin in PBS. The fixed specimens were dehydrated through an ethanol series, cleared in xylene, and embedded in Paraplast Plus^®^ medium (Sigma-Aldrich Co., St. Louis, MO, USA). Tissue slices from the embedded specimens were cut into 4-μm thick sections and stained with hematoxylin and eosin.

### *In situ* hybridization

*In situ* hybridization of sections was carried out as described previously^23^,^67^. Briefly, sagittal 4-μm thick sections were used for *in situ* hybridization. Probes for *Dr-nanos* and *Drpiwi-2* were synthesized from the clone *Dr_sW_028_K12* containing the *nanos* gene^23^ and the clone *Dr_sW_011_L16* containing the *piwi* gene^40^. DIG (digoxigenin) was detected using an alkaline phosphatase-conjugated anti-DIG (digoxigenin) antibody (Roche, Mannheim, Germany) and stained (blue) with NBT/BCIP (17 μg/mL NBT (nitro-blue tetrazolium chloride), 8.8 μg/mL BCIP (5-bromo-4-chloro-3-indolylphosphate, toluidine-salt)) (Roche, Mannheim, Germany).

### Immunohistochemistry

The worms were relaxed in 2% cold HCl (v/v) in 5/8 Holtfreter’s solution for 5 min, and fixed in 4% paraformaldehyde and 2% glutaraldehyde in 5/8 Holtfreter’s solution at RT for 3 h. The worms were dehydrated through a graded ethanol series, embedded in paraffin, sectioned to 4-μm thickness, and mounted on slides. After deparaffinization, the mounted specimens were rinsed with PBS, incubated with 0.5% sodium borohydride in PBS for 20 min at RT, and rinsed three times with PBS containing 0.5% Triton X-100 at RT for 20 min. Tissue sections were then incubated in blocking buffer (10% goat serum in PBTx (PBS containing 0.1% Triton X-100)) for 30 min at RT and then with the appropriate antiserum diluted with blocking buffer at 4 °C overnight. The following day, the sections were rinsed three times with PBTx at RT for 10 min and incubated with the appropriate secondary antibody diluted with blocking buffer at RT for 3 h. They were then rinsed three times in PBTx at RT for 10 min, and the slides were dipped in 1 μg/mL Hoechst 33342 (Sigma, St. Louis, MO, USA) to stain the nuclei. The slides were then rinsed with PBS, mounted with Vectashield (Vector Laboratories, Burlingame, CA), and observed under a fluorescence microscope (Zeiss Axioplan 2; Carl Zeiss, Thornwood, NY). The antibodies used in this study were as follows: anti-conjugated Trp rabbit polyclonal antibody (diluted 1:50; Advanced Targeting Systems, San Diego, CA), normal rabbit immunoglobulin G (1 mg/mL) (diluted 1:50; Sigma-Aldrich, St Louis, MO), and Alexa Fluor 488 conjugated goat anti-rabbit immunoglobulin G (diluted 1:500; Molecular Probes, Eugene, OR).

### Statistical analysis

Data pertaining to the occurrence of worms with a pair of ovaries were analysed using the chi-square test.

With respect to the ovary-inducing activity (%) by treatment with commercial ᴅ-Trp or ʟ-Trp, the dependence of the effects (*Y*) on the doses of ᴅ-Trp or ʟ-Trp (*X*) was analysed on the basis of the following equation.


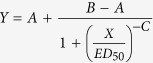


*A, B*, and *C* are defined as the basal value, maximal value, and Hill coefficient, respectively. ED_50_ was calculated by non-linear regression using an in-house program.

## Additional Information

**How to cite this article:** Kobayashi, K. *et al*. The identification of ᴅ-tryptophan as a bioactive substance for postembryonic ovarian development in the planarian *Dugesia ryukyuensis. Sci. Rep.* **7**, 45175; doi: 10.1038/srep45175 (2017).

**Publisher's note:** Springer Nature remains neutral with regard to jurisdictional claims in published maps and institutional affiliations.

## Supplementary Material

Supplementary Information

## Figures and Tables

**Table 1:** Estimation of Trp content in each Fr. M0 + M10 from 4-g samples (wet weight).

Samples	Estimated amount of Trp (μg)
*Bdellocephala brunnea*	2,160
Chicken liver	480
*Dugesia ryukyuensis* (sexual worm)	7,120
*Dugesia ryukyuensis* (asexual worm)	280

**Table 2:** Ovary-inducing activity in the asexual test worms fed with commercial ᴅ-Trp or ʟ-Trp.

Foods	Trp content administered (μg/worm/day)	Number of worms that developed ovaries	Statistical significance^†^
ʟ-Trp	0	3.9% (3/76)	
	0.3	26.7% (20/75)	*P* < 0.001
	1.5	49.0% (24/49)	*P* < 0.001
	3	43.2% (32/74)	*P* < 0.001
ᴅ-Trp	0	2.4% (2/82)	
	0.1 × 10^−4^	7.1% (6/84)	n.s.
	0.1 × 10^−3^	35.4% (29/82)	*P* < 0.001
	0.1 × 10^−2^	40.5% (34/84)	*P* < 0.001
	0.1 × 10^−1^	48.8% (41/84)	*P* < 0.001
	0.1	45.7% (37/81)	*P* < 0.001

^†^Significance was calculated using the chi-square test.
