# Supplementary material for: The identification of ᴅ-tryptophan as a bioactive substance for postembryonic ovarian development in the planarian Dugesia ryukyuensis
Source: Sci Rep. 2017 Mar 24;7:45175. doi: 10.1038/srep45175 (PMC5364533; doi:10.1038/srep45175)
Supplement: Supplementary Information [file srep45175-s1.pdf]

Title*: The identification of D-tryptophan as a bioactive substance for postembryonic ovarian development in the planarian *Dugesia ryukyuensis

***Authors*:** Kazuya Kobayashi[a,*], Takanobu Maezawa[b], Hiroyuki Tanaka[c], Hiroyuki Onuki[d,1], Yurie Horiguchi[e], Hiroshi Hirota[d,2], Tetsuo Ishida[c,3], Kihachiro Horiike[c], Yasutoshi Agata[c], Manabu Aoki[f], Motonori Hoshi[f] and Midori Matsumoto[f]

***Author affiliations*:**

[a]Department of Biology, Faculty of Agriculture and Life Science, Hirosaki University, 3 Bunkyo-cho, Hirosaki, Aomori 036-8561, Japan

[b]Advanced Science Course, Department of Integrated Science and Technology, National Institute of Technology, Tsuyama College, 624-1 Numa, Tsuyama, Okayama 708-8509, Japan

[c]Department of Biochemistry and Molecular Biology, Shiga University of Medical Science, Tsukiwa-cho, Seta, Ohtsu, Shiga 520-2192, Japan

[d]RIKEN Genomic Sciences Center, 1-7-22 Suehiro-cho, Tsurumi-ku, Yokohama 230-0045, Japan

[e]Center for Integrated Medical Research, School of Medicine, Keio University, 35 Shinano-machi, Shinjuku-ku, Tokyo 160-8582, Japan

[f]Department of Biosciences and Informatics, Keio University, 3-14-1 Hiyoshi, Kouhoku-ku, Yokohama 223-8522, Japan

[*]***Corresponding authors*:**

Kazuya Kobayashi

Department of Biology, Faculty of Agriculture and Life Science, Hirosaki University, 3 Bunkyo-cho, Hirosaki, Aomori 036-8561, Japan

Tel & Fax: 81-172-39-3587 E-mail: kobkyram@hirosaki-u.ac.jp

Supplementary Information:

Fig. S1
Legend of Fig. S1

Fig. S2
Legend of Fig. S2

Fig. S3
Legend of Fig. S3

Table S1

Materials and Methods

Fig. S1

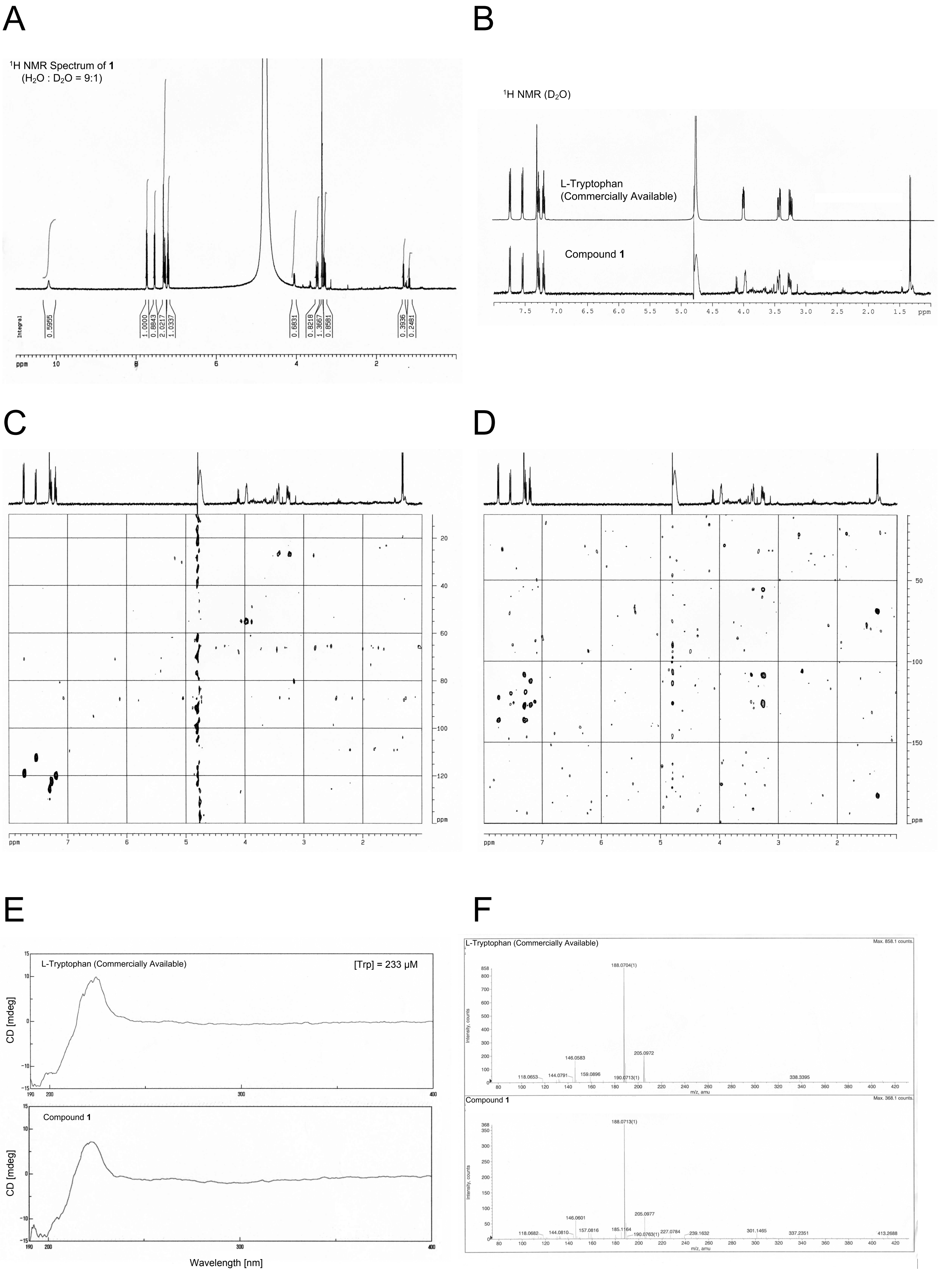

A
1H NMR Spectrum of 1
(H2O : D2O = 9:1)
Integral
0.5955
1.0000
0.8843
2.0217
1.0337
0.6831
0.8218
1.3667
0.8581
0.3936
0.2481
ppm
B
1H NMR (D2O)
L-Tryptophan
(Commercially Available)
Compound 1
ppm
C
D
E
L-Tryptophan (Commercially Available)
[Trp] = 233 μM
CD [mdeg]
Compound 1
Wavelength [nm]
F
L-Tryptophan (Commercially Available)
Max. 858.1 counts.
188.0704(1)
146.0583
205.0972
118.0653
144.0791
159.0896
190.0713(1)
338.3395
Compound 1
Max. 368.1 counts.
188.0713(1)
146.0601
205.0977
118.0682
144.0810
157.0816
185.1164
190.0763(1)
227.0784
239.1632
301.1465
337.2351
413.2688
Intensity, counts
m/z, amu

Fig. S1. Spectral data of compound 1 from Q1 obtained using RESOURCE Q. **(A)** ^{1}H NMR spectrum of compound **1** in H_2O-D_2O (9:1), **(B)** ^{1}H NMR spectra of compound **1** and commercially available L-Trp in D_2O. δ10.17 (br), 7.75 (d, 1H, *J* = 8 Hz), 7.54 (d, 1H, *J* = 8 Hz), 7.31 (s, 1H), 7.29 (dd, 1H, *J* = 8, 8 Hz), 7.20 (dd, 1H, *J* = 8, 8 Hz), 4.04 (dd, 1H, *J* = 8, 5 Hz), 3.42 (dd, 1H, *J* = 15, 5 Hz), 3.24 (dd, 1H, *J* = 15, 8 Hz); **(C)** Hetero-nuclear multiple quantum coherence (HMQC) spectrum of compound **1** in D_2O, **(D)** Hetero-nuclear multiple-bond connectivity (HMBC) spectrum of compound **1** in D_2O. ^{13}C NMR (D_2O) δ176.0 (s), 136.7 (s), 127.3 (s), 125.5 (d), 123.0 (d), 120.4 (d), 118.6 (d), 112.8 (d), 108.4 (s), 54.8 (d), 26.4 (t); **(E)** CD spectra of compound **1** and commercially available L-Trp. CD (H_2O) 223 nm ($\Delta\varepsilon$ + 6.4); **(F)** Electrospray ionization–mass spectrometry (ESI-MS)/MS spectra of compound **1** and commercially available L-Trp. ESI-MS/MS *m*/*z* 205, 188, 146.

Fig. S2

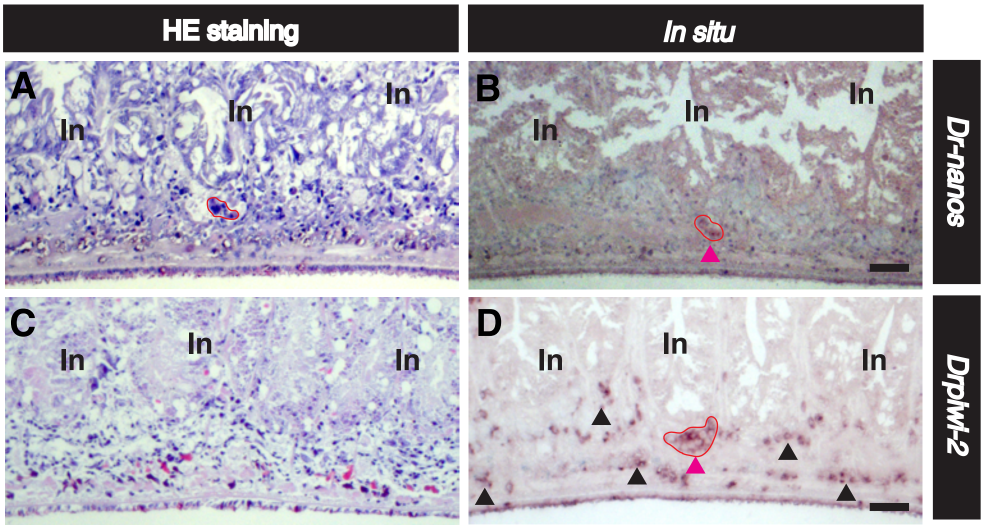

HE staining
In situ
A
In
In
In
B
In
In
In
Dr-nanos
C
In
In
In
D
In
In
In
Drpiwi-2

Fig. S2. Expression of *Dr-nanos* and *Drpiwi-2* in tissue surrounding ovary development sites in asexual worms. (A–D) Tissue areas encompassed by the red line are cell masses of female PGCs (ovarian primordia). The dorsal sides are located at the top of images. In: intestine. Scale bar: 50 μm. Two successive serial paraffin sections were used for staining with HE and *in situ* hybridization with each other. **(A, C)** HE staining. **(B)** *In situ* hybridization of *Dr-nanos*. **(D)** *In situ* hybridization of *Drpiwi-2*. The ovarian primordia displaying *in situ* hybridization signal are indicated by magenta arrowheads. *Drpiwi-2* is expressed in neoblasts as well as in germline cells [40]. In asexual worms, equal levels of *Drpiwi-2* expression are detected in both ovarian primordia and neoblasts (indicated by black arrowheads). Typical ovarian primordia were not identified in the HE image **(C)**.

Fig. S3

A

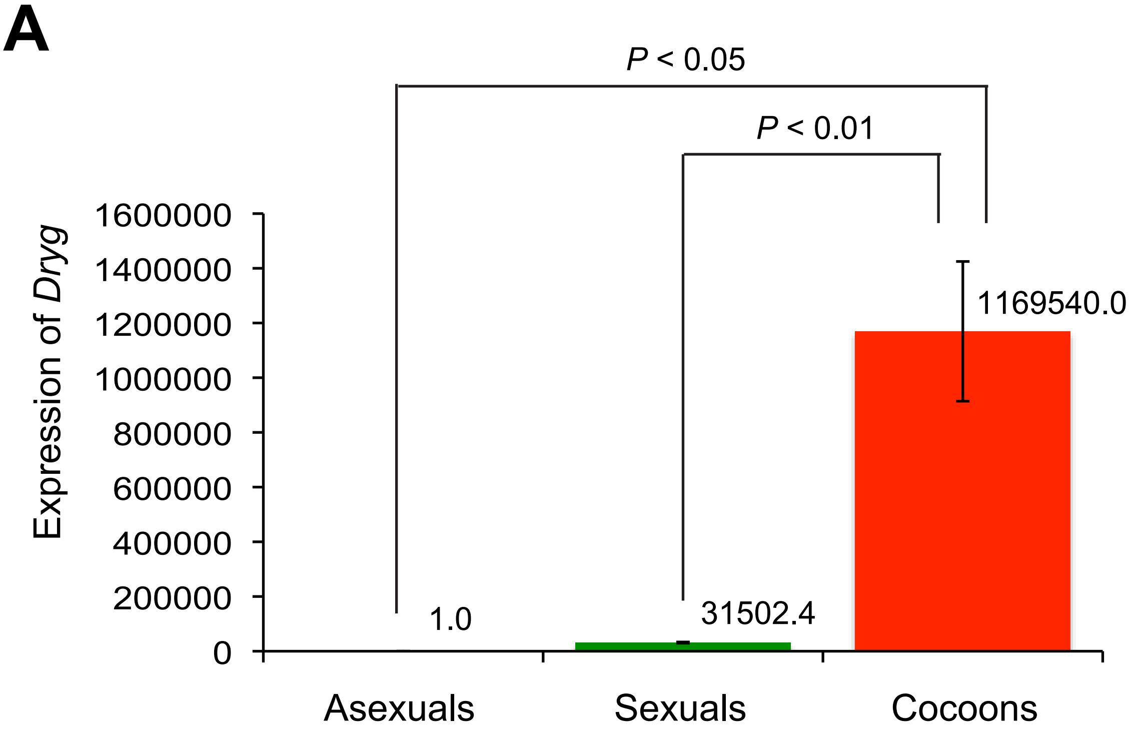

P < 0.05
P < 0.01
Expression of Dryg
1600000
1400000
1200000
1000000
800000
600000
400000
200000
0
1169540.0
31502.4
1.0
Asexuals
Sexuals
Cocoons

B

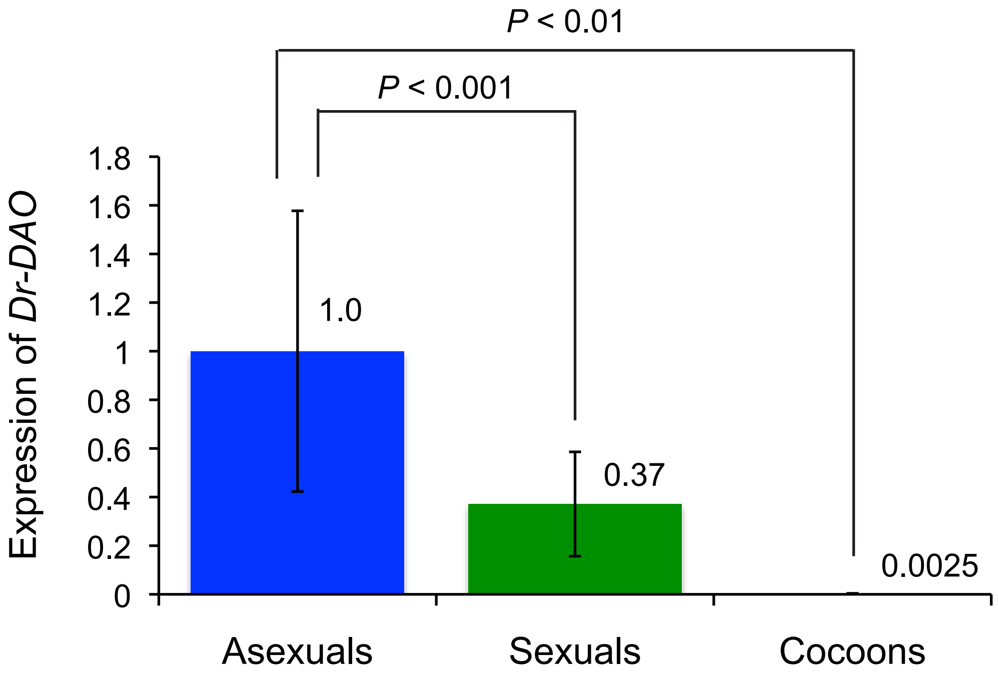

P < 0.01
P < 0.001
Expression of Dr-DAO
1.8
1.6
1.4
1.2
1
0.8
0.6
0.4
0.2
0
1.0
0.37
0.0025
Asexuals
Sexuals
Cocoons

Fig. S3. Quantitative reverse transcription polymerase chain reaction (qRT-PCR) analysis of *Dryg* and *Dr-DAO* expression in asexual worms, sexual worms, and cocoons of *D. ryukyuensis*. qRT-PCR of **(A)** *Dryg*, a marker gene for yolk gland cells was performed in asexual worms of the OH strain, sexual worms of the OH strain, and their cocoons just after deposition, and **(B)** *Dr-DAO*, a *DAO* (D-amino acid oxidase) homolog gene was also analyzed. Expression values are represented as the average of three asexual worms, three sexual worms, and three cocoons, respectively (± S.E.). The results represent relative expression in the asexual worms. Statistical significance was calculated using a *t*-test.

Table S1. Relative amounts of each proteinogenic amino acid in asexual worms, sexual worms, and cocoons.

Amino acid	Relative area			Sexual/Asexual	Cocoons/Asexual
	Asexual	Sexual	Cocoons		
Trp	1.40E-02	2.30E-01	6.10E-01	16.4	43.6
Tyr	2.20E-02	1.60E-01	6.50E-02	7.3	3.0
Cys	1.10E-03	3.00E-03	N.D.*	2.7	N.D.*
Gln	4.10E-02	7.30E-02	1.20E-02	1.8	0.3
Asp	1.30E-01	2.00E-01	9.50E-02	1.5	0.7
Thr	2.60E-02	3.60E-02	4.20E-02	1.4	1.6
Ala	1.30E-01	1.80E-01	2.60E-02	1.4	0.2
Asn	3.20E-02	4.10E-02	2.30E-02	1.3	0.7
Pro	2.40E-02	3.00E-02	1.20E-02	1.3	0.5
Ile	6.60E-02	7.60E-02	1.70E-02	1.2	0.3
His	9.00E-02	1.00E-01	7.80E-02	1.1	0.9
Phe	4.80E-02	5.20E-02	8.10E-02	1.1	1.7
Lys	8.90E-02	9.40E-02	4.60E-02	1.1	0.5
Ser	5.50E-02	5.70E-02	3.50E-02	1.0	0.6
Gly	2.10E-02	2.20E-02	6.10E-03	1.0	0.3
Leu	9.10E-02	9.20E-02	1.80E-02	1.0	0.2
Val	8.30E-02	8.10E-02	1.50E-02	1.0	0.2
Arg	8.50E-02	7.60E-02	2.40E-02	0.9	0.3
Glu	2.40E-01	1.90E-01	2.70E-02	0.8	0.1
Met	1.60E-02	9.30E-03	1.60E-03	0.6	0.1

*Not detected.

Materials and Methods

Quantitative reverse transcription-polymerase chain reaction (qRT-PCR)

Besides the visual evaluation of sex-inducing activity, we used qRT-PCR to analyze the expression of marker genes required for sexual induction. qRT-PCR was performed using the 7300 Real-Time PCR System (Applied Biosystems, Foster City, CA). Each reaction mixture (25 μL) contained 12.5 μL of Power SYBR Green PCR Master mix (Applied Biosystems, Foster City, CA), 0.4 μM of gene-specific primers, and 0.5 μL of the cDNA template. The PCR reaction was as follows: 50°C for 2 min and 95°C for 10 min, followed by 40 cycles of 95°C for 15 s and 60°C for 1 min. Measurements were normalized to the expression levels of the elongation factor 1 alpha homolog (*Dr-ef1a*). The gene-specific primers used were as follows: *Dr-ef1a/DrC_00434*, 5′ -TTGGTTATCAACCCGATGGTG-3′ and reverse, 5′ -TCCCATCCCTTGTACCATGAC-3′ [64]; *Dr-DAO* forward, 5′ -TCGCTACCAATCCAAAGCATC-3′ and reverse, 5′ -CGGCTGTTTCCCACAATTTC-3′ [50]; *Dryg* forward, 5′ -AAATCTATCGTTGCCCGATG-3′ and reverse, 5′ -TCGCATCGTTTTGATGTTTG-3′ [47].

Quantitative analysis of amino acids

Asexual worms, sexual worms, and fresh cocoons of *Dugesia ryukyuensis* were used for quantitative analysis of proteinogenic amino acids (Human Metabolome Technologies Inc., Tsuruoka, Japan). Asexual worms (30 mg wet weight, 5 worms), sexual worms (36 mg wet weight, 3 worms), and fresh cocoons (30 mg wet weight, 10 cocoons) were used. Frozen samples were transferred into 1,500 μL of 50% aqueous acetonitrile (v/v) containing 20 μM of the external standard. After

homogenization using BMS-M10N21 equipment (BMS, Tokyo) at 1,500 rpm for 120 s on ice performed three times, the suspensions were centrifuged at 2,300 g for 5 min at 4°C. The resultant supernatants were ultrafiltered using a Millipore Ultrafree-MC PLHCC HMT Centrifugal Filter Device, 5 kDa (Millipore, Billerica, MA). The filtrates were then dried and dissolved in 50 μL of ultrapure water. The samples obtained were then subjected to capillary electrophoresis time-of-flight mass spectrometry (CE-TOFMS) analysis using the Agilent CE-TOFMS system (Agilent Technologies, Santa Clara, CA) at 4°C. The detected peaks were aligned according to their m/z values and normalized migration times. The peaks were mean-centered and scaled using their standard deviations on a per-peak basis as a pretreatment. The relative area, m/z, and retention time (RT) of each peak were obtained using the software MasterHands ver.2.16.0.15 (Keio University, Japan). Each peak corresponding to a proteinogenic amino acid was identified on the basis of m/z and RT. The relative area of each peak corresponding to a proteinogenic amino acid was used for comparison of the asexual worms, the sexual worms, and the fresh cocoons.
